# Virtual application of in situ simulation during a pandemic

**DOI:** 10.1017/cem.2020.375

**Published:** 2020-04-24

**Authors:** Erich Hanel, Monika Bilic, Kelly Hassall, Mary Hastings, Farah Jazuli, Michael Ha, Brendon Trotter, Cory Fraser, Greg Rutledge

**Affiliations:** * St. Joseph's Healthcare Hamilton, Hamilton, ON; † Department of Family Medicine, Division of Emergency Medicine, McMaster University, Hamilton, ON; ‡ Michael G. DeGroote School of Medicine, McMaster University, Hamilton, ON; § Department of Medicine, Division of Emergency Medicine, McMaster University, Hamilton, ON

**Keywords:** Pandemic, simulation, video

## Abstract

The coronavirus disease 2019 (COVID-19) pandemic introduced challenges to the use of simulation, including limited personal protective equipment and restricted time and personnel. Our use of video for in situ simulation aimed to circumvent these challenges and assist in the development of a protocol for protected intubation and simultaneously educate emergency department (ED) staff. We video-recorded a COVID-19 respiratory failure in situ simulation event, which was shared by a facilitator both virtually and in the ED. The facilitator led discussions and debriefs. We followed this with in situ run-throughs in which staff walked through the steps of the simulation in the ED, handling medications and equipment and becoming comfortable with use of isolation rooms. This application of in situ simulation allowed one simulation event to reach a wide audience, while allowing participants to respect social distancing, and resulted in the education of this audience and successful crowdsourcing for a protocol amidst a pandemic.

## Résumé

L'arrivée de la pandémie causée par la maladie à coronavirus 2019, COVID-19, a eu pour effet de rendre plus difficile le recours à la simulation, en raison notamment du port de l'équipement de protection individuelle et des restrictions de temps et de personnel. L'enregistrement vidéo d'une simulation sur place visait à contourner ces difficultés et à faciliter l'élaboration d'un protocole d'intubation protégée, en plus d'offrir de la formation au personnel du service des urgences (SU). Pour ce faire, nous avons procédé à l'enregistrement vidéo d'une simulation, sur place, d'un cas d'insuffisance respiratoire causée par la COVID-19, vidéo qui a été partagée par un animateur, aussi bien virtuellement qu'au SU. Celui-ci a dirigé les discussions et les rétroactions. Ont suivi des répétitions sur place dans lesquelles le personnel franchissait les étapes de la simulation au SU, manipulant les médicaments et le matériel nécessaires et se familiarisant avec le travail dans les salles d'isolement. Cette application de simulation sur place a permis non seulement de joindre un grand nombre de personnes, mais aussi de rendre possibles le respect de la distanciation physique et la formation des participants, sans compter la réussite d'une externalisation à grande échelle de l'élaboration d'un protocole en pleine période de pandémie.

## BACKGROUND

Previous work has demonstrated the utility of in situ simulation to advance health care provider skills and aid in the development of protocols and procedures.^1,2^ However, the use of simulation under the constraints inflicted by a pandemic has not yet been addressed. The coronavirus disease 2019 (COVID-19) pandemic posed new challenges to the execution of in situ simulation in the emergency department (ED), as well as time-sensitive pressures for the development of protected code blue and intubation protocols. Challenges included restricted time, personnel, and personal protective equipment (PPE), with changing guidelines on appropriate use. These factors were addressed together through an uncommon application of in situ simulation with mixed online and offline distribution.

### Purpose

The aim of this innovation was: (1) to develop a protocol for protected intubation with input from front-line interprofessional teams under significant stress; and (2) to educate ED staff during a pandemic.

### Description

**Figure 1.**
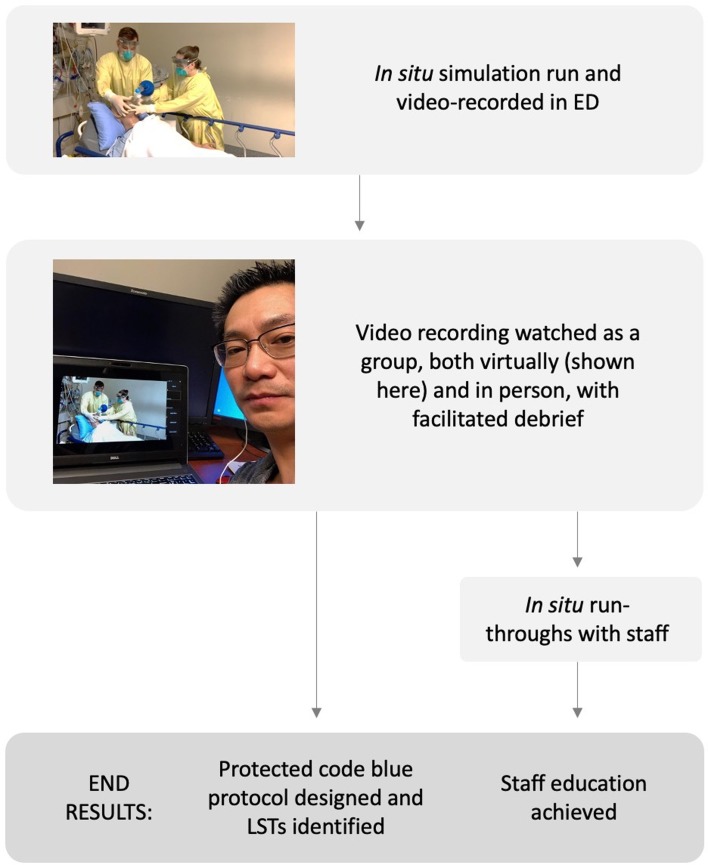
Schematic depicting the described process from simulation to the development of the protected code blue protocol and staff education.

The multidisciplinary simulation team at a busy ED in Hamilton, Ontario, had previously facilitated weekly in situ simulation within the ED. Our experience has shown that this in situ simulation was feasible and productive in a busy ED.^3^ However, the aforementioned difficulties of the pandemic challenged this, requiring the application of a different style of simulation, one which used video recording and sharing (Figure 1). This team designed a case of respiratory failure secondary to COVID-19 infection and carried out the simulation at a time where ED volume was low, in the so-called “lull” of the COVID-19 pandemic. Participants were individuals who were on shift at the time. They were given a short prebrief (which included the design of the simulation session), and consent for video recording was obtained. Facilitators ran and recorded the simulation in a negative pressure room with associated anteroom. The simulation was unrehearsed and executed in real time, such that it was imperfect and designed to elicit debrief discussions from viewers about where the process could be improved.

Over the following 14 days, the 9-minute video was shared in small groups both electronically (which allowed for participants to respect social distancing), as well as in the emergency department. The video was not released for independent review. Facilitators provided a pre-amble (explaining that this is not meant to be a model simulation and addressing the vulnerability of video participants), presented the video, and led a debrief. The debriefing mechanism asked participants what they saw, what could be improved, and how the simulation compared with current protected code blue protocols. The facilitator was able to provide education around points as needed. The protected code blue process and intubation protocol was designed by the education team in tandem to the virtual simulations. Following the debrief, an electronic survey was distributed by means of email to allow further anonymous feedback and to gauge team resilience and preparedness.

This was complemented by additional in situ simulations on site to capture staff from different departments, including nursing, respiratory therapy, and clinical medicine. These were a run through of the simulation in real time, without manikins or PPE, with team members working together verbalizing procedures done in the context of isolation rooms and anterooms, and collecting required equipment. This allowed increased ED staff participation in the simulation after viewing the video with minimal expense, enhancing their educational experience and familiarity with proper use of negative pressure rooms and the locations of required equipment and medications.

## DISCUSSION

Video-based learning has been used previously in the classroom, but its use remotely in the same manner as an actual in situ event has not been reported.^4^ The described application of simulation consisted of video-recording an in situ simulation event, which allowed for protocol development and staff education, with discussions of patient management during this pandemic. There was significant staff participation at the leadership level from all departments (registered nurses, respiratory therapists, and physicians). With the previous regular in situ simulations, there was feedback that this format was intimidating to mixed groups with varying levels of experience. These formats of virtual video-based simulation offered perhaps the most protected safe environment. This pilot project of education delivery using an in situ model has now branched out to include simulation educators across the region who have trialed a similar model.

This model also allowed the teams to identify and modify site-specific latent safety threats (LSTs), which are system-based threats to patient safety that are not previously recognized.^5^ As an example, one such LST identified was the difficulty with clear communication between the patient room and the anteroom. This was identified in the initial simulation and debrief and echoed in video-related debriefs as well. This led to introduction of baby monitors to allow for clearer two-way communication between the two rooms. This was tested in isolation and improvements were made based on feedback from the run-through sessions, such as having a dedicated provider at the monitor to relay complex requests such as infusion medication orders.

Using the debriefs and postsimulation surveys, we checked-in with staff and elicited their perceptions surrounding the pandemic. The survey revealed that more than half of the respondents were concerned about resource limitation, particularly PPE. Other concerns included the risk of infection to health care providers and their families, and the notion of being unprepared for the pandemic. Given these legitimate concerns, it is essential that simulation continues to be used, despite these difficult times. The utility of in situ simulation in increasing preparedness and allowing for the creation and improvement of protocols was leveraged as part of this virtual simulation method. In a time of uncertainty, this method allowed for the protocol to evolve daily and respond to challenges immediately, in parallel with the pandemic situation. This method offered a means of rapid knowledge dissemination, using very few resources and saving time, PPE, and simulation equipment, while allowing for social distancing and eliminating geography as a limitation to education delivery.

## SUMMARY

We used a video-recorded simulation with virtual distribution to aid in the development of a protected code blue protocol, incorporating staff input and education under the constraints and pressures of a global pandemic.
